# Encoding integers and rationals on neuromorphic computers using virtual neuron

**DOI:** 10.1038/s41598-023-35005-x

**Published:** 2023-07-06

**Authors:** Prasanna Date, Shruti Kulkarni, Aaron Young, Catherine Schuman, Thomas Potok, Jeffrey Vetter

**Affiliations:** 1 Oak Ridge National Laboratory, Oak Ridge, TN 37830 USA; 2 University of Tennessee, Knoxville, TN 37996 USA

**Keywords:** Computational science, Computer science, Information technology, Software, Applied mathematics

## Abstract

Neuromorphic computers emulate the human brain while being extremely power efficient for computing tasks. In fact, they are poised to be critical for energy-efficient computing in the future. Neuromorphic computers are primarily used in spiking neural network–based machine learning applications. However, they are known to be Turing-complete, and in theory can perform all general-purpose computation. One of the biggest bottlenecks in realizing general-purpose computations on neuromorphic computers today is the inability to efficiently encode data on the neuromorphic computers. To fully realize the potential of neuromorphic computers for energy-efficient general-purpose computing, efficient mechanisms must be devised for encoding numbers. Current encoding mechanisms (e.g., binning, rate-based encoding, and time-based encoding) have limited applicability and are not suited for general-purpose computation. In this paper, we present the virtual neuron abstraction as a mechanism for encoding and adding integers and rational numbers by using spiking neural network primitives. We evaluate the performance of the virtual neuron on physical and simulated neuromorphic hardware. We estimate that the virtual neuron could perform an addition operation using just 23 nJ of energy on average with a mixed-signal, memristor-based neuromorphic processor. We also demonstrate the utility of the virtual neuron by using it in some of the *μ*-recursive functions, which are the building blocks of general-purpose computation.

## Introduction

Neuromorphic computers perform computations by emulating the human brain^1^. Akin to the human brain, they are extremely energy efficient in performing computations^2^. For instance, while CPUs and GPUs consume around 70–250 W of power, a neuromorphic computer such as IBM’s TrueNorth consumes around 65 mW of power, (i.e., 4–5 orders of magnitude less power than CPUs and GPUs)^3^. The structural and functional units of neuromorphic computation are neurons and synapses, which can be implemented on digital or analog hardware and can have different architectures, devices, and materials in their implementations^4^. Although there are a wide variety of neuromorphic computing systems, we focus our attention on spiking neuromorphic systems composed of these neurons and synapses. Spiking neuromorphic hardware implementations include Intel’s Loihi^5^, SpiNNaker2^6^, BrainScales2^7^, TrueNorth^3^, and DYNAPS^8^. These characteristics are crucial for the energy efficiency of neuromorphic computers. For the purposes of this paper, we define neuromorphic computing as any computing paradigm (theoretical, simulated, or hardware) that performs computations by emulating the human brain by using neurons and synapses to communicate with binary-valued signals (also known as spikes).

Neuromorphic computing is primarily used in machine learning applications, almost exclusively by leveraging spiking neural networks (SNNs)^9^. In recent years, however, it has also been used in non-machine learning applications such as graph algorithms, Boolean linear algebra, and neuromorphic simulations^10–12^. Researchers have also shown that neuromorphic computing is Turing-complete (i.e., capable of general-purpose computation)^13^. This ability to perform general-purpose computations and potentially use orders of magnitude less energy in doing so is why neuromorphic computing is poised to be an indispensable part of the energy-efficient computing landscape in the future.

Neuromorphic computers are seen as accelerators for machine learning tasks by using SNNs. To perform any other operation (e.g., arithmetic, logical, relational), we still resort to CPUs and GPUs because no good neuromorphic methods exist for these operations. These general-purpose operations are important for preprocessing data before it is transferred to a neuromorphic processor. In the current neuromorphic workflow—preprocessing on CPU/GPU and inferencing on neuromorphic processor—more than 99% of the time is spent in data transfer (see Table 7). This is highly inefficient and can be avoided if we do the preprocessing on the neuromorphic processor. Devising neuromorphic approaches for performing these preprocessing operations would drastically reduce the cost of transferring data between a neuromorphic computer and CPU/GPU. This would enable performing all types of computation (preprocessing as well as inferencing) efficiently on low-power neuromorphic computers deployed on the edge. To develop efficient approaches for *preprocessing* data on a neuromorphic processor, we must first have an efficient mechanism for *encoding* data on the neuromorphic processor.

One of the biggest limitations of neuromorphic computing today is the inability to encode data efficiently^14^. Although there are several studies on the performance of neural network models with low precision representations of parameters such as weights^15^, these approximate representations are not suitable for general-purpose computing. Several methods can encode numbers on neuromorphic computers^16^. However, their scope is restricted to the specific application for which they were designed and is not suitable for general-purpose computation. Furthermore, no good mechanism exists for encoding negative integers and positive and negative rational numbers on neuromorphic computers. The ability to encode basic data types such as numbers, letters, and symbols is vital for any computing platform. Efficient mechanisms for encoding rational numbers would significantly expand the scope of neuromorphic computing to new application areas such as non-SNN-based machine learning (regression, support vector machines), a wide range of graph and network problems, general-purpose computing applications, linear and non-linear optimization, simulation of physical systems, and perhaps even finding good solutions to NP-complete problems. Working with rational numbers is central to these areas, and neuromorphic mechanisms for efficiently encoding rational numbers would enable us to solve these problems in an energy-efficient manner.

To this extent, we present the *virtual neuron* abstraction for addressing the limitation of neuromorphic computers to encode numbers. This is the first step toward performing general-purpose computations on neuromorphic computers. To the best of our knowledge, the virtual neuron numerical representation is the first encoding mechanism that can efficiently encode positive and negative integers and rational numbers on a neuromorphic computer. Specifically, our main contributions are as follows:

1. We introduce the virtual neuron as a new primitive element abstraction, which is made up of spiking neurons and synapses. The virtual neuron primitive accepts two input values of variable precision and outputs the summation. The values are interpreted using a spatial encoding mechanism that leverages the binary representation of numbers to encode positive and negative integers and rational numbers as a collection of neurons. We also discuss the computational complexity of the virtual neuron. See section “The virtual neuron.”
2. We implement the virtual neuron in the Neural Simulation Technology (NEST) simulator^17^ and on the Caspian neuromorphic simulator^18^ and test its performance when adding 8-, 16-, and 32-bit rational numbers. This is outlined in the “Implementation details and methods” and “Testing results” sections.
3. We analyze the performance of the virtual neuron on neuromorphic hardware by analyzing the run time using a digital neuromorphic hardware design (Caspian), and we estimate the energy usage of the virtual neuron on a mixed-signal memristor-based hardware design (mrDANNA)^19^. This is presented in the “Testing results” section.
4. We demonstrate the usability of the virtual neuron abstraction by using it to implement five functions: constant function, successor function, predecessor function, multiply by $-1$ function, and *N*-neuron addition. This is covered in the “Applications” section. Without the virtual neuron, implementing these functions on a neuromorphic computer would be extremely challenging.

## Related work

Neuromorphic computing was introduced by Carver Mead in the 1980s^20^. Since then, it is primarily used for SNN-based machine learning applications, including computer vision^21^, natural language^22,^ and speech recognition^23^. These applications are mainly found in embedded systems, edge computing, and Internet of Things (IoT) devices because they have strict requirements for size, weight, and power^24–26^. Several on-chip and off-chip learning algorithms that leverage gradient-based and local learning rules have been suggested for training SNNs in neuromorphic applications^27–30^. Neuromorphic computing has also been used in neuroscience simulations^31^. These simulations span a wide range of neuron and synapse models, the most popular of which is the leaky-integrate-and-fire (LIF) neuron model^32^. Our virtual neuron will use spiking neurons that are of the LIF type as well. The latest additions to the arsenal of neuromorphic computing applications include graph algorithms^10,33,34^, autonomous racing^35^, epidemiological simulations^12^, classifying supercomputer failures^36^, $\mu$-recursive functions^13^, and Boolean matrix-vector multiplication^11^. For designing neuromorphic algorithms, a theoretical framework for determining the computational complexity has also been proposed^37^.

Most of the above applications are based on binary numbers and Boolean arithmetic. This is largely due to the spiking behavior of the neuron—the spikes can be interpreted as a 1, whereas lack of spike can be interpreted as a 0. This spiking behavior naturally lends itself to binary or Boolean operations. Leveraging this behavior, several mechanisms for encoding numbers (mainly positive integers) have been proposed in the literature. Choi et al. propose a neuromorphic implementation of hypercolumns, including mechanisms for encoding images^38^. Cohen et al. use neuromorphic methods to classify images that have been encoded as spikes^39^. Hejda et al. present a mechanism for encoding image pixels as rate-coded optical spike trains^40^. Sengupta and Roy encode neural and synaptic functionalities in electron spin as an efficient way to perform neuromorphic computation^41^. Yi et al. propose a field programmable gate array (FPGA) platform to be used as a spike-time-dependent encoder and dynamic reservoir in neuromorphic computers^42^.

Iaroshenko and Sornborger propose neuromorphic mechanisms that use the two’s complement for encoding numbers and performing arithmetic and matrix operations^43^. However, their approach uses numbers of neurons and synapses that are of the quadratic and cubic order, respectively. Lawrence et al. perform neuromorphic matrix multiplication by using an intermediate transformation matrix for encoding that is flattened into a neural node^44^. Schuman et al. propose three ways of encoding positive integers on neuromorphic computers, which are in turn used in many different applications^16^. Zhao et al. develop a compact, low-power, and robust spiking-time-dependent encoder, designed with a LIF neuron cluster and a chaotic circuit with ring oscillators^45^. Zhao et al. develop a method for representing data using spike-time dependent encoding that efficiently maps a signal’s amplitude to a spike time sequence that represents the input data^46^. Zhao et al. propose an analog temporal encoder for making neuromorphic computing robust and energy efficient^47^. Wang et al. use radix encoding of spikes to realize SNNs more efficiently and improve the speedup by reducing the overall latency for machine learning applications^48^. Plank et al. make an effort to realize basic computations on neuromorphic platforms that leverage the inherent structure and parameters of SNNs for logic operations (e.g., AND, OR, and XOR)^49^. George et al. perform IEEE 754 compliant addition with SNNs by designing a system based on the Neural Engineering Framework (NEF) and implement, simulate, and test the design using Nengo^50^. This approach uses an ensemble of 300 neurons to represent each bit, and the function of each component in the adder is approximated with NEF to determine the appropriate synapse weights. Dubey et al. extend this work to perform IEEE 754 compliant multiplication using the same encoding method and a similar methodology of using NEF to approximate the functions of the multiplier subcomponents^51^.

Most of these encoding mechanisms can encode binary or Boolean numbers, and some can encode positive integers as well. These methods are designed with specific applications in mind (e.g., image applications), and it is not clear if they can be used for general-purpose neuromorphic computation, in which arithmetic operations must be performed on positive and negative integers/rationals. Moreover, some of the encoding mechanisms such as binning tend to lose information by virtue of discretization. To the best of our knowledge, an efficient mechanism for encoding positive and negative rational numbers exactly does not exist in the neuromorphic literature. We address this gap by proposing the virtual neuron. In our quest for general-purpose, energy-efficient neuromorphic computing, being able to encode rational numbers efficiently and exactly is a critical milestone.

## Neuromorphic computing model

Neuromorphic computing systems implement vastly different neuron and synapse models, and the precise model details depend on the specific hardware implementation. We leverage the neuromorphic computing model described in previous work^13,37^. This model can be realized by all of the most commonly used neuron (e.g., LIF neurons, Izhikevich neurons) and synapse (e.g., synapses with and without synaptic plasticity) models in the literature by appropriately setting their parameters.

**Figure 1:**
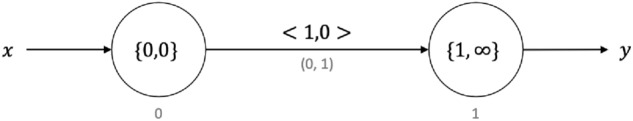
Symbolic notation describing a neuromorphic circuit with two neurons: 0 and 1. Neuron 0 has a threshold of 0 and a leak of 0 and is hence indicated as 0, 0. Neuron 1 has a threshold of 1 and leak of $\infty$, indicated by $\{1, \infty \}$. The synapse is described as $\langle {1, 0}\rangle$, which indicates weight of value 1 and a delay of 0.

**Figure 2:**
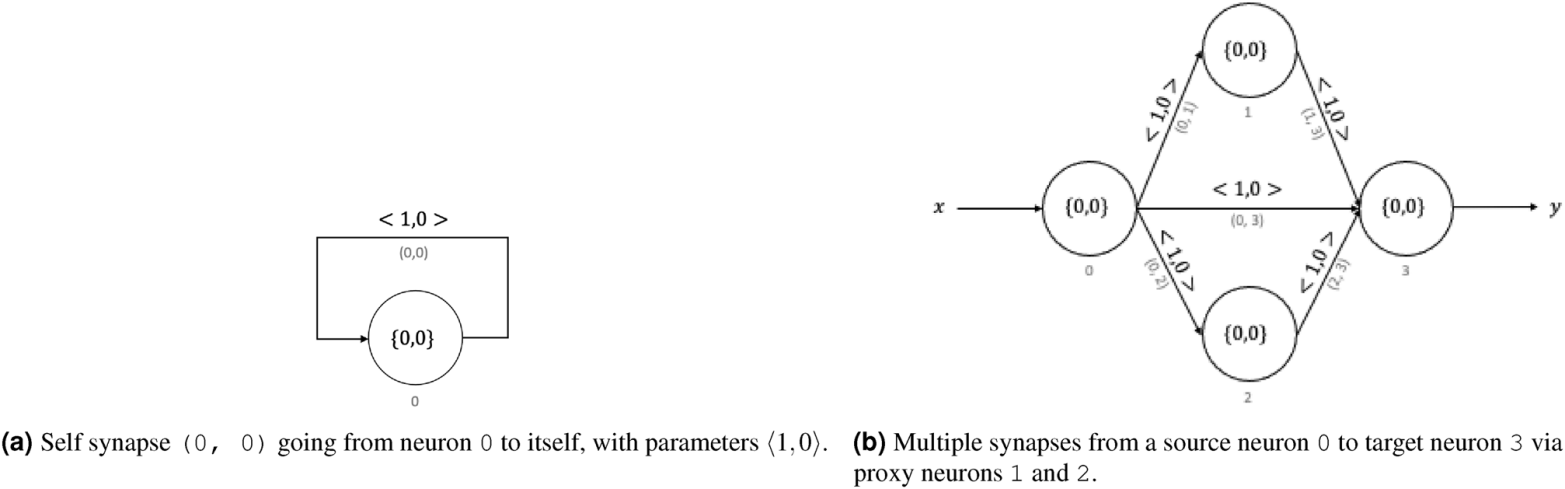
Different types of connections supported by the virtual neuron.

### Structural components

Our model of neuromorphic computing is composed of two structural components: neurons and synapses. A neuron is the fundamental unit from which a neuromorphic circuit is built. A synapse constitutes a connection from a pre-synaptic (source) neuron to a post-synaptic (target) neuron. We further assume that all-to-all connectivity between neurons is possible. Neurons could also receive signals from, and send signals to, external synapses, which are used for data I/O. Such neurons are called I/O neurons.

Figure 1 shows the symbolic notation for neurons, synapses, and neuromorphic circuits throughout this paper. The circles represent neurons, and arrows represent synapses. Neurons are referenced by using the whole numbers shown below them. Synapses are referenced by using a tuple that contains the references of the pre-synaptic neuron and the post-synaptic neuron. External synapses do not have explicit references. Figure 1 shows an external synapse on the left feeding an input *x* to the input neuron 0. Neuron 0 is connected to neuron 1 via the synapse (0, 1). Neuron 1 returns the output *y* through the external synapse on the right. We allow different types of synaptic connections between two neurons: self-synapse (autapses) and multiple synapses (multapses) as used in several types of neuromorphic models and neuroscience models^52^ (Fig. 2). Figure 2a shows a self-synapse to the neuron 0. We do not allow a pre-synaptic and a post-synaptic neuron to be directly connected via multiple synapses. Such a functionality could be achieved by routing a signal through multiple proxy neurons as shown in Fig. 2b. Notably, in our current work on the virtual neuron, we do not use these self-connections or multiple connections between two neurons; however, such models can be employed in future applications of the virtual neuron if needed.

### Functional aspects and parameters

The neurons in our model are based on the LIF neurons. They accumulate signals from incoming synapses in their internal state until a threshold is reached. After reaching the threshold, they spike and send their signal via outgoing synapses. The neurons reset to an internal state given by the reset state after spiking. Our neurons have a leak, which specifies the time it takes to push their internal state back to the default internal state in case they do not spike. For instance, neurons with a leak of 0 (instantaneous leak) would have no recollection of their internal state. On the other hand, neurons with infinite leak would remember their internal state exactly until they spike and reset. We denote the internal state of the *i-*th neuron using the English letter $v_i$. The two neuron parameters—threshold and leak—are denoted by the Greek letters $\nu _i$ and $\lambda _i$. They are assumed to be whole numbers. We use braces to specify neuron parameters $\{\nu _i, \lambda _i\}$ in our circuit diagrams. In Fig. 1, neuron 0 has a threshold of 0 and a leak of 0, as indicated by $\{0, 0\}$; neuron 1 has a threshold of 1 and a leak of $\infty$, as indicated by $\{1, \infty \}$.

Synapses in our model receive signals from their pre-synaptic neuron, multiply the signal by their weight, stall for a time indicated by their delay, and deposit the signal in their post-synaptic neuron. For a synapse connecting neuron i to neuron j, its weight and delay are denoted by $\omega _{i,j}$ and $\delta _{i,j}$. The weights are integer-valued and the delays are whole numbers. In our circuit diagrams, we indicate the synaptic parameters using chevrons, $\langle {\omega _{i,j}, \delta _{i,j}}\rangle$, on top of the synapses. For instance, the synapse (0, 1) in Fig. 1 has a weight of 1 and delay of 0, and its parameters are indicated by $\langle {1, 0}\rangle$. External synapses do not have weights or delays.

We assume it takes one unit of time for a spike to travel across any synapse in the absence of delay. This assumption is fundamental to determining the computational complexity of neuromorphic algorithms. Under this assumption, the smallest value for a synaptic delay is 1. A neuromorphic algorithm is defined by the configuration of a neuromorphic circuit. We assume that a neuromorphic circuit interfaces with data that is also neuromorphic (i.e., encoded as spikes). The outputs of a neuromorphic circuit are represented by the spiking behavior of the neurons. The spikes have an associated time at which they occur and a neuron on which they occur. The output of the circuit is called the spike raster, which enumerates the time and neuron for each spike that occurs over the course of the circuit’s operation.

## The virtual neuron

**Figure 3:**
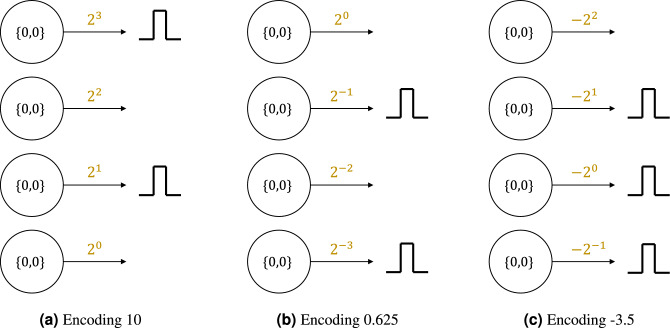
Encoding mechanism of the virtual neuron. Numbers can be encoded by selecting the appropriate synaptic weights. Here, we use four neurons to encode (**a**) positive integers; (**b**) positive rationals; and (**c**) negative rationals. The four spiking neurons can encode 4 bits of information.

**Figure 4:**
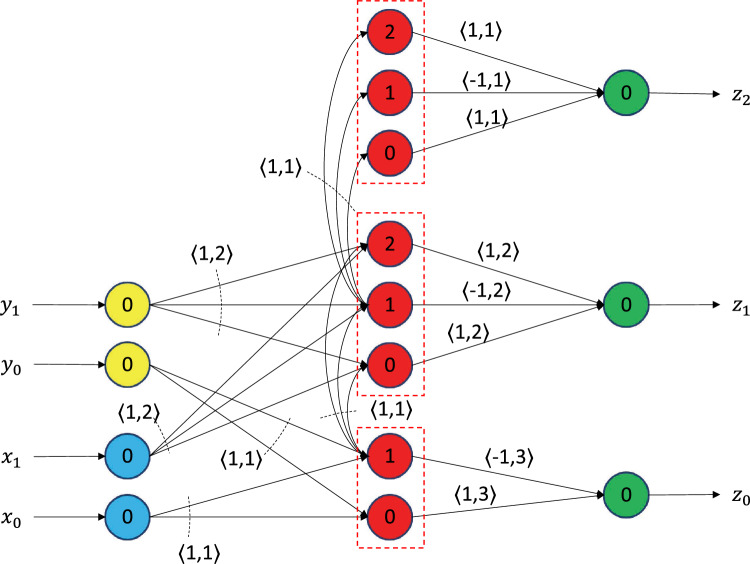
A 2-bit virtual neuron. Takes two 2-bit numbers as input on the left, *X* and *Y*, represented as $x_1$, $x_0$ and $y_1$, $y_0$, respectively. Adds the two numbers and generates their sum on the right. The sum of two 2-bit numbers can at most be a 3-bit number.

**Figure 5:**
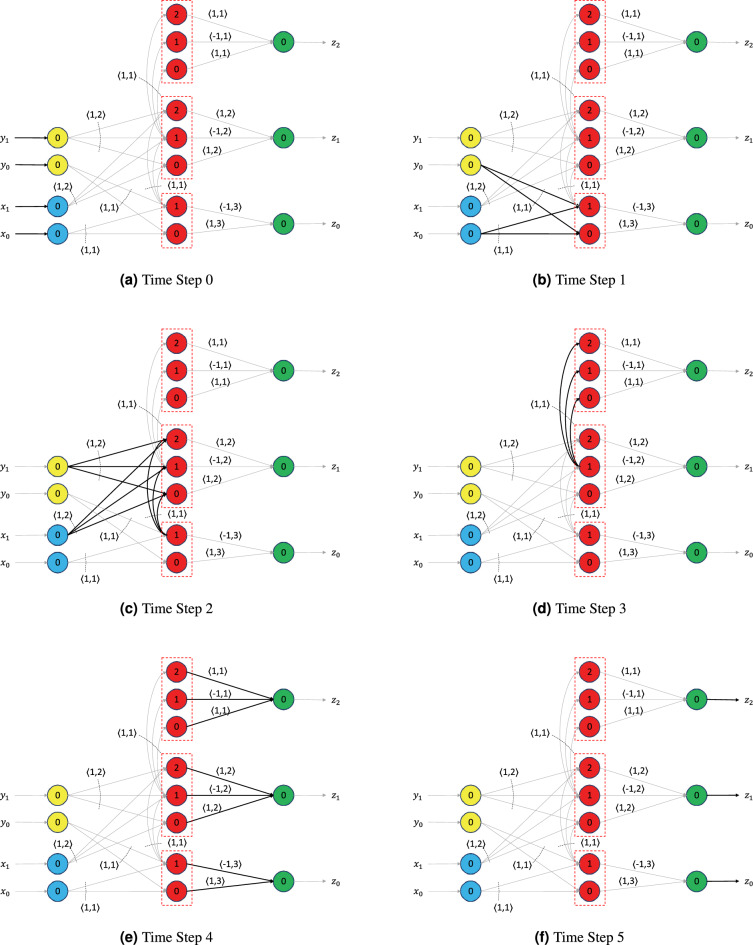
Working mechanism of the virtual neuron. (**a**) Time Step 0: Input signals are received in the input neurons (blue and yellow). (**b**) Time Step 1: Signals from input neurons representing the least significant bits (bottom blue and bottom yellow) are received in the first set of bit neurons, which also represent the least significant bit. (**c**) Signals from input neurons and first set of bit neurons are received in the second set of bit neurons. (**d**) Signals from second set of bit neurons are received in the third set of bit neurons. (**e**) Signals from all bit neurons are received in the output neurons. (**f**) Output neurons return the output of the circuit.

Virtual neuron is a new SNN primitive that is structurally composed of groups of LIF neurons and synapses that are connected in a particular way. Functionally, the virtual neuron mimics the behavior of a numerical artificial neuron with identity activation. In other words, the virtual neuron takes two input values and adds them together for the output. The encoding mechanism used by the virtual neuron allows groups of LIF neurons to be interpreted as positive or negative rational numbers that are then added together similarly to a ripple carry adder^53^. The rationale behind the encoding mechanism of the virtual neuron is rooted in the binary encoding of numbers. Figure 3 shows three ways of encoding 4-bit numbers on a neuromorphic computer. Notice that each neuron in the figure represents a bit. The synapse coming out of the neuron assigns a value to the binary spike of the neuron by multiplying it with its synaptic weight. By having powers of 2 as the synaptic weights, we can encode rational numbers using a group of neurons. For instance, the synapses coming out of the four neurons in Fig. 3a have weights of $2^0$, $2^1$, $2^2$, and $2^3$. When the second and fourth neurons (from the bottom) spike, the result gets multiplied by 2 and 8 in the outgoing synapses, respectively. This is interpreted as the number 10 under this encoding mechanism. Similarly, we can set the synaptic weights to be negative powers of 2, as shown in Fig. 3b. This enables us to encode positive fractions as well. When the first and third neurons (from the bottom) spike as shown in the figure, the result is interpreted as 0.625. Lastly, if the synaptic weights are set to negatives of positive and negative powers of 2 as shown in Fig. 3c, then we can encode negative rational numbers. When the three neurons spike in the figure, the output is interpreted as $-3.5$.

We now show how the virtual neuron can integrate the incoming signals and generate a rational number as output. For ease of explanation, we stick to the 2-bit virtual neuron as shown in Fig. 4. The 2-bit virtual neuron takes as input two 2-bit numbers, *X* and *Y*, shown in the figure as $[x_1, x_0]$ (blue neurons) and $[y_1, y_0]$ (yellow neurons), respectively. It then adds *X* and *Y* in the three groups of bit neurons, which are shown in red. We call them bit neurons because they are responsible for the bit-level operations in the circuit (e.g., bitwise addition, propagating the carry bit). Finally, it produces a 3-bit number, *Z,* as output, shown in the figure as $[z_2, z_1, z_0]$ (green neurons).

The default internal states of all neurons are set to $-1$. Furthermore, all neurons have a leak of 0, and this means they reset to their default internal state instantaneously if they do not spike. The reset state (or reset voltage) of all neurons is set to $-1$ so that the internal state of all neurons will be reset to $-1$ after they spike. The numbers on the neurons indicate their thresholds. For example, the top bit neurons (red neurons) have thresholds 0, 1, and 2, respectively. The synapse parameters are indicated in chevrons on the top or bottom of the synapses. The first parameter is the synaptic weight, and the second parameter is the synaptic delay. If a group of synapses has the same parameters, then it is indicated with a dotted arc. The synaptic delays are adjusted such that the bit operations of red neurons are synchronized, and the output *Z* is produced at the same time.

Next, we describe the inner workings of the virtual neuron shown in Fig. 4 by taking the example $[x_1, x_0] = [1, 1]$, and $[y_1, y_0] = [0, 1]$. We start our analysis when the inputs *X* and *Y* have been received in the blue and yellow neurons—let us call this the 0-th time step, as shown in Fig. 5a. In the first time step (Fig. 5b), the bottom set of bit neurons in red receive an input of 1 along each of their incoming synapses. Thus, the total incoming signal at both these neurons is 2, which changes their internal state from $-1$ to 1. As a result, both the bottom red neurons spike. Their spikes are sent along their outgoing synapses, which delay the signal for 3 time steps.

In the second time step (Fig. 5c), the middle group of bit neurons receives all of the inputs: 1 from the blue incoming neuron representing $x_1$, 0 from the yellow neuron representing $y_1$, and 1 from the bit neuron with a threshold of 1 in the bottom group. Thus, the sum of their incoming signals is 2, and their internal states reach a value of 1. As a result, neurons with thresholds 0 and 1 in the middle group of bit neurons spike, whereas the one with threshold 2 does not spike. The spikes from the middle red neurons with thresholds 0 and 1 are sent to the green output neuron representing $z_1$ along their outgoing synapses, which stall for 2 time steps.

In the third time step (Fig. 5d), the 3-bit neurons in the top group of red neurons receive an input of 1 along each of their incoming synapses. As a result, their internal states are incremented by 1 to the value of 0. The neuron with a 0 threshold spikes as a result and sends its spike along its outgoing synapse to the green neuron representing $z_2$.

In the fourth time step (Fig. 5e), the green neurons representing $z_0$, $z_1$, and $z_2$ receive their inputs. $z_0$ receives a 1 and $-1$ from the bit neurons with the thresholds 0 and 1, respectively, in the bottom group of red neurons. Its total input is thus $1 - 1 = 0$, which keeps its internal state at $-1$, and it does not spike. Similar operations happen at the green neuron representing $z_1$. It too does not spike. The green neuron representing $z_2$ receives a signal of 1 from the bit neuron with the threshold of 0 in the top red set. As a result, its internal state is incremented by 1 to the value of 0, and it spikes.

At the fifth time step (Fig. 5f), the net output $[z_2, z_1, z_0]$ from the circuit is [1, 0, 0], which can be interpreted as a 4 in binary. Given that our inputs were $[x_1, x_0] = [1, 1]$ and $[y_1, y_0] = [0, 1]$ (i.e., $X = 3$, and $Y = 1$), we have received the correct output of 4 from the virtual neuron circuit. Although we restricted ourselves to 2-bit positive integers in this example, we show in the subsequent subsections that similar circuits can be used to encode and add two rational numbers in the virtual neuron and generate a rational number as output. Finally, note that we did not use powers of 2 in the synapses inside of the virtual neuron. However, the powers of 2 are used implicitly to interpret the value of the input and output groups, and care must be taken to connect the virtual neurons together such that the representation is maintained. Depending on the application, powers of 2 as synaptic weights may be used on the incoming or outgoing synapses for a given virtual neuron. As an example, the synaptic weights can be used on the outgoing synapse to accumulate the numerical value in a traditional LIF neuron.

In the following subsections, we present virtual neuron circuits that have higher precision. We let $P_+$ and $P_-$ denote the number of bits used to represent positive and negative numbers, respectively. We call them positive precision and negative precision. In general, the positive precision, $P_+$, will be distributed among bits used to represent positive integers ($2^0, 2^1, 2^2, \ldots$) and positive fractionals ($2^{-1}, 2^{-2}, 2^{-3}, \ldots$). Similarly, the negative precision, $P_-$, will be distributed among bits used to represent negative integers ($-2^0, -2^1, -2^2, \ldots$) and negative fractionals ($-2^{-1}, -2^{-2}, -2^{-3}, \ldots$).

We now describe the connections for a virtual neuron with arbitrary precision. Each input neuron has both threshold and leak set as 0. Each input $x_i$ and $y_i$ is connected to the set of bit neurons that correspond to bit *i*. In the case of bit 0, there are two such bit neurons, while for every other bit, there are three neurons per bit, shown in red. The synaptic weights of all these connections are unity, and their delays are $i+1$. Each set of bit neurons has neurons with thresholds of 0 and 1. All bit neurons except the 0 bit have a neuron with a threshold of 2 as well. The neuron with a threshold of 1 in the set of neurons that represents bit *i* is connected to all neurons in the $(i+1)$-th set. This neuron is responsible for propagating the carry bit to the next set of bit neurons. It spikes only when there is a carry operation to be performed at the *i*-th bit. The carry synapses have both weights and delays as unity. The bit neurons of the *i*-th bit are connected to the *i*-th output neuron. The synaptic weights for the bit neurons having thresholds of 0 and 2 are 1, whereas those for the bit neurons having threshold of 1 are $-1$. The $-1$ weight is seen as an inhibitory connection that cancels the signal coming from the neuron with threshold 0 in the same bit set. The delays on the synapses going from *i*-th bit set to the *i*-th output neuron are set to $\max \{P_+, P_-\} - i + 1$. This delay ensures that all output neurons spike at the same time.

**Figure 6:**
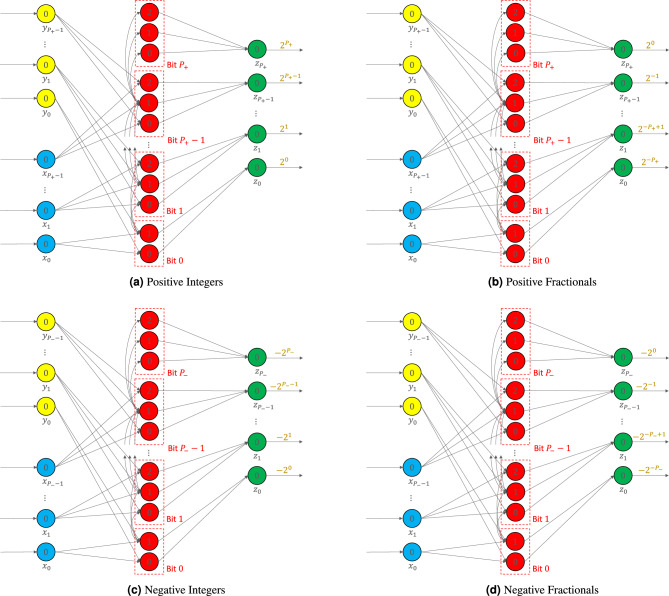
Encoding (**a**) positive integers, (**b**) positive fractionals, (**c**) negative integers, and (**d**) negative fractionals using the virtual neuron. The key differences are in the synaptic weight values of the outgoing synapses (i.e., synapses coming out of the green neurons located on the right in each figure). Positive integers (**a**) and positive fractionals (**b**) have $P_+$ precision, whereas negative integers (**c**) and negative fractionals (**d**) have $P_-$ precision.

### Positive integers

Figure 6a shows the virtual neuron circuit that takes two $P_+$ bit numbers *X* and *Y* as inputs, shown as blue and yellow neurons, respectively. The bit-level addition and carry operations are performed by the bit neurons shown in red. There are $P_+ + 1$ groups of these bit neurons. Finally, the output of the virtual neuron *Z* has $P_+ + 1$ bit precision and is shown by the green output neurons. In the figure, we omit synapse parameters for brevity. Note that the synaptic weights on the outgoing synapses are positive powers of 2.

### Positive fractionals

Figure 6b shows the $P_+$ bit virtual neuron for encoding positive fractionals. The circuit is almost identical to Fig. 6a. The only difference is in the synaptic weights of the outgoing synapses. In this case, these synapses have negative powers of 2 (i.e., $2^0, 2^{-1}, 2^{-2}, 2^{-3}, \ldots$) as their weights.

### Negative integers

Figure 6c shows the virtual neuron circuit for encoding negative integers. It takes two $P_-$ bit numbers *X* and *Y* as inputs. After standard virtual neuron operations, a $P_- + 1$ bit number *Z* is produced as the output. In this case, these weights are negatives of positive powers of 2 (i.e., $-2^0, -2^1, -2^2, \ldots$).

### Negative fractionals

Figure 6d shows the $P_-$ bit virtual neuron circuit for encoding negative fractionals. This circuit is identical to Fig. 6c, except the outgoing synapses have weights that are negatives of negative powers of 2 (i.e., $-2^0, -2^{-1}, -2^{-2}, \ldots$).

### Positive and negative rational numbers

**Figure 7:**
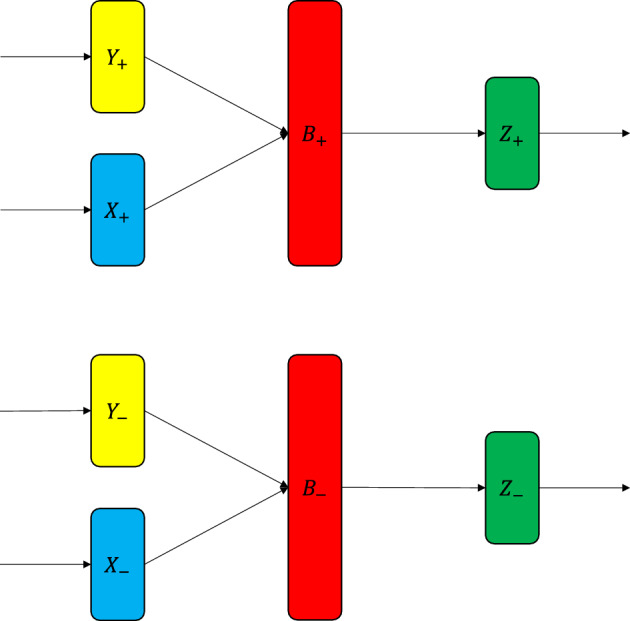
Encoding $P_+$ bit positive rationals and $P_-$ bit negative rationals using the virtual neuron.

In this case (Fig. 7), the virtual neuron operates on two $P_+ + P_-$ bit rational numbers *X* and *Y* as inputs. These are shown in blue and yellow rectangles, which denote aggregation of respective neurons. The positive precision $P_+$ is split between the positive integers and positive fractionals. Similarly, negative precision is split between the negative integers and negative fractionals. Note that the positive part of the circuit (upper half) is completely independent from the negative part of the circuit (lower half).

### Computational complexity

**Table 1:** Neurons, synapses, and time taken with increasing precision.

Number of bits of positive precision ($P_+$)	Number of neurons ($6P_++3$)	Number of synapses ($12P_+$)	Time steps for virtual neuron operations ($P_+ + 2$)
1	9	12	3
2	15	24	4
4	27	48	6
8	51	96	10
16	99	192	18
32	195	384	34
64	387	768	66
128	771	1536	130

This table only shows scalability for $P_+$. However, the same type of scalability can be expected for $P_-$.

**Figure 8:**
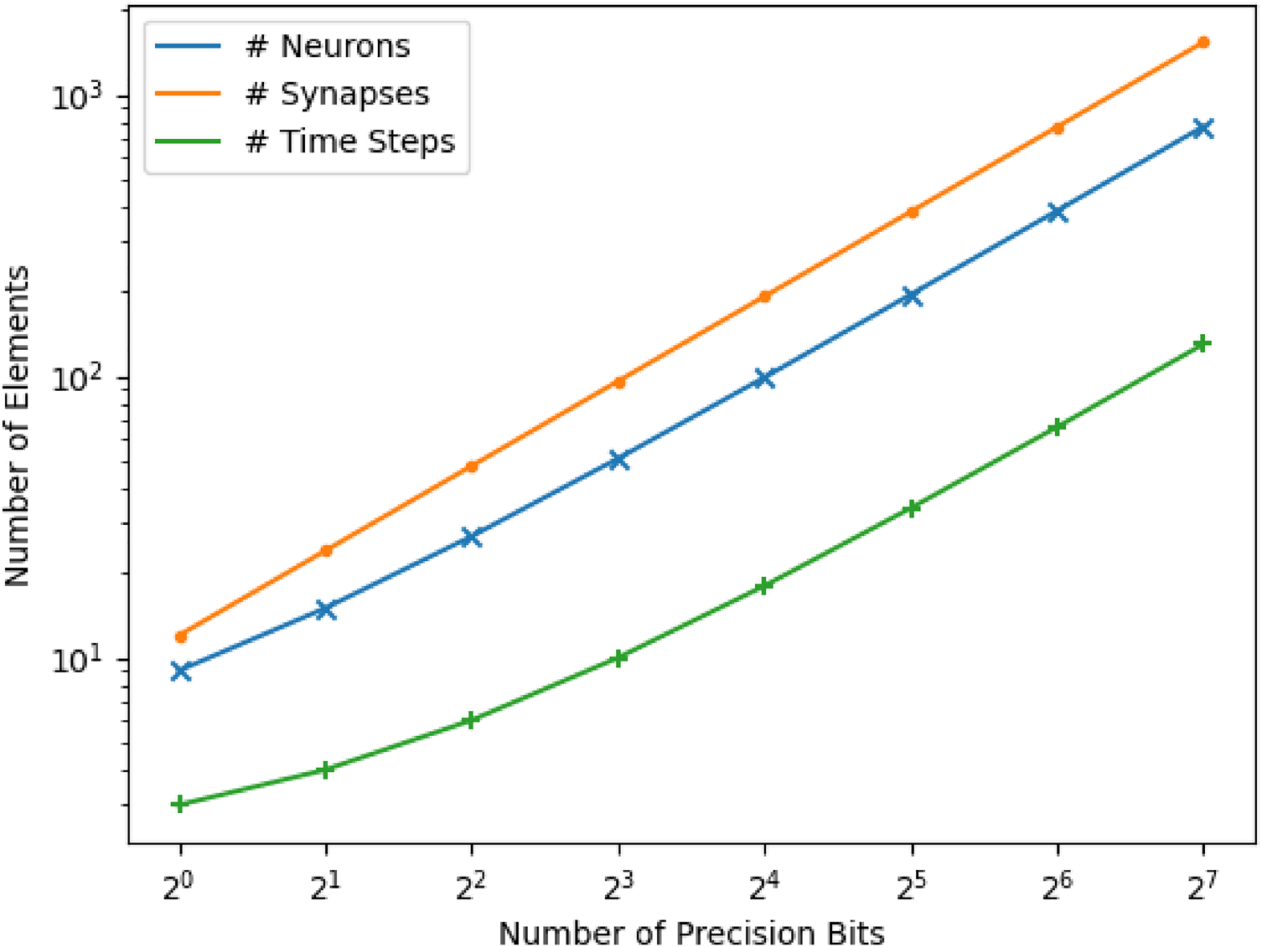
Scalability of the number of neurons, synapses, and time steps for the number of precision bits ($P_+$ or $P_-$).

Appendix shows the Python code used to set up the virtual neuron using the NEST simulator. This code includes creating neurons with specific neuron parameters and then connecting them using synapses, which have their own set of parameters. For $P_+$ bit positive operations, we use $\mathscr {O}(P_+)$ neurons and synapses and perform the virtual neuron operations in $\mathscr {O}(P_+)$ time steps. Similarly, for $P_-$ bit negative operations, we use $\mathscr {O}(P_-)$ neurons and synapses and perform the virtual neuron operations in $\mathscr {O}(P_-)$ time steps. All in all, we use $\mathscr {O}(P_+ + P_-)$ neurons and synapses and consume $\mathscr {O}(\max \{P_+, P_-\})$ time steps for the virtual neuron operations.

**Table 2:** Comparing the virtual neuron to other neuromorphic encoding mechanisms for representing two *N*-bit numbers exactly.

Metrics	Binning^16^	Rate^16^	Time^16^	Virtual neuron	IEEE-754^50,51^
Time complexity	$\mathscr {O}$(1)	$\mathscr {O}$($2^N$)	$\mathscr {O}$($2^N$)	$\mathscr {O}$(1)	$\mathscr {O}$(1)
# Neurons	$\mathscr {O}$($2^N$)	$\mathscr {O}$(1)	$\mathscr {O}$(1)	$\mathscr {O}$(N)	Ensembles of *N* neurons with dimension and radius properties$^1$ $\mathscr {O}$(N)$^2$
Accuracy	100%	100%	100%	100%	$>90$% at 500 neurons per bit. Encoded error at 0 with $>300$ Neurons per bit.
Energy efficiency (# of spikes in the worst case)	$\mathscr {O}$(1) spikes	$\mathscr {O}$($2^N$) spikes	$\mathscr {O}$(1) spikes	$\mathscr {O}$(N) spikes; N spikes	Energy results unpublished
Energy efficiency (# spikes in the average case)	$\mathscr {O}$(1) spikes	$\mathscr {O}$($2^N$) spikes	$\mathscr {O}$(1) spikes	$\mathscr {O}$(N) spikes; N/2 spikes	Energy results unpublished

$^1$Dimension refers to the number of values represented by the ensemble (for a scalar quantity, this is 1). Radius defines the range of values that can be represented by the ensemble. For the cited work, dimension is 1, and radius is set to 2.

$^2$Authors only looked at IEEE floating point, so how the representation scales with numerical precision is unclear.

We validate these space and time complexities empirically for positive operations by increasing $P_+$. The results of this analysis apply to negative operations as well. We increase the positive precision from $1, 2, 4, \ldots , 128$ and count the number of neurons, synapses, and time steps in each case. The numerical results are presented in Table 1. These numbers are also plotted in Fig. 8 on logarithmic *X* and *Y* axes. From the table, we can conclude that we use $6P_+ + 3$ neurons, $12P_+$ synapses, and $P_+ + 2$ time steps for virtual neuron operations.

**Table 3:** Comparing the virtual neuron to other neuromorphic encoding mechanisms for adding two *N*-bit numbers.

Metrics	Binning^16^	Rate encoding^16^	Virtual neuron	IEEE 754^50^
Time to solution	$\mathscr {O}$(1)	$\mathscr {O}$($2^N$)	$\mathscr {O}$(N)	Constant
# Neurons	$\mathscr {O}$($2^N$)	$\mathscr {O}$(1)	$\mathscr {O}$(N)	3075
# Synapses	$\mathscr {O}$($2^N$)	$\mathscr {O}$(1)	$\mathscr {O}$(N)	N/A
Energy efficiency (# spikes in the worst case)	$\mathscr {O}$($2^N$)	$\mathscr {O}$($2^N$)	$\mathscr {O}$(N)	N/A
Energy efficiency (# spikes in the average case)	$\mathscr {O}$($2^N$)	$\mathscr {O}$($2^N$)	$\mathscr {O}$(N)	N/A
Accuracy	100%$^1$	Depends on model type	100%	100% with 300 neurons per bit

$^1$Accuracy is bound by the synapse weight and accumulation accuracy.

We can extend these time complexities to negative operations to conclude that they would require $6P_- + 3$ neurons, $12P_-$ synapses, and $P_- + 2$ time steps. This validates the space complexity as needing $\mathscr {O}(P_+ + P_-)$ neurons and synapses. Because the positive and negative operations happen in parallel, the overall time complexity of the circuit would stem from the larger of $P_+$ and $P_-$. So, the overall time complexity is validated as $\mathscr {O}(\max \{P_+, P_-\})$.

Lastly, in computing the above space and time complexities, our assumption is that the positive and negative precisions are variable. However, we envision using the virtual neuron in settings where a neuromorphic computer has a fixed, predetermined positive and negative precision. This is similar to how the precision on our laptops and desktops is fixed to 32, 64, or 128 bits. In such a scenario, $P_+$ and $P_-$ can be treated as constants. Thus, the resulting space and time complexities for the virtual neuron would all be $\mathscr {O}(1)$.

Table 2 compares different neuromorphic encoding approaches in the literature with our approach using the virtual neuron. Because a neuromorphic computer consumes energy that is proportional to the number of spikes, we use the number of spikes in the worst and average case as an estimate for the energy usage of different neuromorphic approaches. Across different comparison metrics (e.g., network size, number of spikes), the virtual neuron scales linearly with the bit-precision *N* while giving the exact representation of the input number. Other approaches take either exponential space (Binning), exponential time (Rate Encoding), or are unable to represent rational numbers exactly (IEEE 754).

Table 3 presents the comparison of computational complexity for performing addition with two *N*-bit numbers under different neuromorphic encoding schemes. Here, we do not include a temporal encoding scheme because under such a simple approach, binary spikes occurring at different time instances cannot be added exactly by spiking neurons. Although the virtual neuron can perform the addition operation in linear time steps and by using a linear number of neurons, synapses, and energy (as estimated by the spiking efficiency), other approaches use either exponential time or exponential space or consume an exponential amount of energy for their operations.

## Implementation details and methods

We implemented the virtual neuron in Python using the NEST simulator. We ran the simulations on a MacBook Pro equipped with an Intel Core i7 quad-core processor running at 2.3 GHz and 32 GB of LPDDR4X memory running at 3733 MHz. We wrote a VirtualNeuron class, the constructor of which took a list-like object of length 4 as the precision vector. The elements of this vector corresponded to the number of bits for positive integers, positive fractionals, negative integers, and negative fractionals. We then computed the positive precision as the sum of the first two elements of the precision vector and computed the negative precision as the sum of the third and fourth elements of the precision vector.

We then created all the neurons and set their parameters correctly. We used the iaf_psc_delta neuron model in NEST. All neurons had an internal state of $-1.0$. In NEST, the internal state corresponds to the voltage of the membrane potential (V_m) parameter. All neurons had a leak of $10^{-6}$, which is a good approximation to 0 leak that we require in our circuits. In NEST, the leak corresponds to the tau_m neuron parameter. All neurons except the bit neurons (red neurons) had a neuron threshold of 0. The group of bit neurons corresponding to the least significant bit in both the positive and negative parts of the circuit had only two neurons with thresholds 0 and 1. All other groups of bit neurons had three neurons with thresholds 0, 1, and 2 respectively. There were $P_+$ ($P_-$) such groups in the positive (negative) part of the circuit, making a total of $P_+ + 1$ ($P_- + 1$) groups of bit neurons corresponding to the $P_+ + 1$ ($P_- + 1$) output bits in the positive (negative) parts of the circuit.

After the neurons were created, we set up the synapses. First, synapses between the positive (negative) incoming neurons and positive (negative) bit neurons were created. These synapses had synaptic weights set as 1.0 and the synaptic delays as *i* + 1, where *i* ranges from 0 to $P_+$ ($P_-$). Second, we set up the carry synapses between the consecutive groups of positive (negative) bit neuron groups. The carry synapses go from the bit neuron having a threshold of 1 in the *i-*th group to all neurons in the $(i+1){\text {-th}}$ group, where *i* goes from 0 to $P_+$ ($P_-$). The carry synapses had both weights and delays set as 1.0. Finally, we set up synapses from groups of positive (negative) bit neurons to their corresponding outgoing neurons. The synapses coming from bit neurons with thresholds 0 and 2 had weights of 1.0, whereas those coming from bit neurons with thresholds 1 had weights of $-1.0$. Furthermore, these synapses in the positive (negative) part of the circuit had delays given by $\max \{P_+, P_-\} - i + 1$ for *i* ranging from 0 to $P_+$ ($P_-$). We also wrote a function, connect_virtual_neurons(A, B, C), that connects three virtual neurons A, B, and C such that A and B serve as inputs to C. The weights and delays on these synapses were all 1.0.

## Test results

We tested our implementation of the virtual neuron on 8-, 16-, and 32-bit rational numbers. The precision vectors fed to the class constructors in each of these cases were [2, 2, 2, 2], [4, 4, 4, 4], and [8, 8, 8, 8], respectively. Next, we generated two numbers within the appropriate precision by generating spikes through the spike_generator in NEST and then sent these spikes to the *X* and *Y* inputs of the virtual neuron. We let the simulation run long enough to receive an output from the virtual neuron’s *Z* output. Last, we checked if the output received from *Z* was indeed the sum of numbers sent to *X* and *Y*. For the results presented in the “8-bit virtual neuron”–“32-bit virtual neuron” and “Applications” sections, we used a desktop computer equipped with an Intel Core i9 8-core CPU running at 3.6 GHz and 64 GB of DDR4 memory running at 2667 MHz.

### 8-bit virtual neuron

**Table 4:** Testing the virtual neuron on 8-bit rational numbers.

$X_+$		$Y_+$		$Z_+ = X_+ + Y_+$		$X_-$		$Y_-$		$Z_- = X_- + Y_-$	
Decimal	Binary	Decimal	Binary	Decimal	Binary	Decimal	Binary	Decimal	Binary	Decimal	Binary
0.75	0011	1.0	0100	1.75	00111	− 2.75	1011	− 2.5	1010	− 5.25	10101
2.5	1010	1.75	0111	4.25	10001	− 3.75	1111	− 0.25	0001	− 4.0	10000
0.25	0001	2.75	1011	3.0	01100	− 2.75	1011	0.0	0000	− 2.75	01011
3.5	1110	3.5	1110	7.0	11100	− 2.5	1010	− 0.25	0001	− 2.75	01011
3.0	1100	3.25	1101	6.25	11001	0.0	0000	− 1.0	0100	− 1.0	00100

Precision is [2, 2, 2, 2] for positive integer, positive fraction, negative integer and negative fraction respectively.

For the 8-bit case, we tested all permutations of the input numbers—a total of 65,536 cases. It took 17,698 s to complete the 65,536 cases in NEST, or approximately 0.27 s per case. The smallest and the largest positive numbers that can be represented in the inputs are 0.0 and 3.75, respectively. The analogous negative numbers that can be represented in the inputs are $-3.75$ and 0.0. The positive outputs can range from 0 to 7.75, and the negative outputs can range from $-7.75$ to 0.0. A randomly selected sample of results is shown in Table 4. As shown, $X_+ + Y_+ = Z_+$, and $X_- + Y_- = Z_-$ for all rows. The binary representations of *X* and *Y* were fed to the input neurons, and the binary representation of *Z* was received as the output of the circuit.

### 16-bit virtual neuron

**Table 5:** Testing the virtual neuron on 16-bit rational numbers.

$X_+$	$Y_+$	$Z_+ = X_+ + Y_+$	$X_-$	$Y_-$	$Z_- = X_- + Y_-$
2.5625	13.3125	15.875	− 11.375	− 6.75	− 18.125
2.3125	11.375	13.6875	− 13.9375	− 9.3125	− 23.25
15.875	1.5625	17.4375	− 2.9375	− 4.6875	− 7.625
8.625	8.9375	17.5625	− 10.1875	− 1.625	− 11.8125
14.6875	11.625	26.3125	− 10.625	− 11.875	− 22.5

Precision is [4, 4, 4, 4] for positive integer, positive fraction, negative integer and negative fraction respectively.

The results from the 16-bit testing are shown in Table 5. The range for positive and negative inputs in this case is [0.0, 15.9375] and $[-15.9375$, 0.0], respectively. The range for positive and negative outputs is [0.0, 31.9375] and $[-31.9375$, 0.0]. In this case, we tested 100,000 permutations of inputs generated uniformly at random. A snippet of the results is shown in Table 5. It took 55,380 s to run these 100,000 cases on the NEST simulator, or 0.55 s per case. One can infer that the virtual neuron is mimicking an artificial neuron with an identity activation function, and that we could encode positive and negative rational numbers on a neuromorphic computer using this approach.

### 32-bit virtual neuron

**Table 6:** Testing the virtual neuron on 32-bit rational numbers.

$X_+$	$Y_+$	$Z_+ = X_+ + Y_+$	$X_-$	$Y_-$	$Z_- = X_- + Y_-$
212.56640625	218.7265625	431.29296875	− 203.421875	− 98.91796875	− 302.33984375
1.375	184.94921875	186.32421875	− 4.36328125	− 92.73046875	− 97.09375
254.3359375	48.87109375	303.20703125	− 134.390625	− 211.43359375	− 345.82421875
44.203125	177.1171875	221.3203125	− 231.0703125	− 207.06640625	− 438.13671875
143.6171875	214.41796875	358.03515625	− 8.1171875	− 224.01953125	− 232.13671875

Precision is [8, 8, 8, 8] for positive integer, positive fraction, negative integer, and negative fraction, respectively.

For the 32-bit case, we generated 100,000 permutations of inputs uniformly at random. The range for positive and negative inputs in the 32-bit case is [0.0, 255.99609375] and $[-255.99609375$, 0.0], respectively. The range for positive and negative outputs is [0.0, 511.99609375] and $[-511.99609375$, 0.0]. Five randomly selected permutations are presented in Table 6. It took 111,978 s to run 100,000 cases in NEST, or approximately 1.12 s per case). Once again, it can be concluded that the virtual neuron can encode and add rational numbers on neuromorphic computers. Furthermore, it can do so exactly and scales linearly with the positive and negative precisions.

### Caspian and hardware testing

We implemented and tested the 16-bit virtual neuron using the Caspian simulator and μCaspian digital FPGA hardware^18^. Because μCaspian does not implement a synaptic delay but instead implements an axonal delay, the virtual neuron implementation was adjusted to use axonal delay instead of synaptic delay. The rest of the structure is the same as the NEST virtual neuron implementation, and the neuron and synapse counts and network time steps to solution are the same as in the NEST implementation.

μCaspian is a digital neuromorphic processor implementation that uses an FPGA. The processor is event-based and processes all the spikes that occur at one time step before moving to the next time step. Time multiplexing of neurons is used to reduce the size of the design. μCaspian is intentionally designed to target the small and low-power iCE40 UP5k FPGA. Because of this, μCaspian only supports up to 256 neurons and 4096 synapses. This is enough to support up to a 32-bit virtual neuron adder; however, to include room for the input and output neurons, we tested it with the 16-bit virtual neuron. The 16-bit virtual neuron test implementation on μCaspian used 152 neurons and 242 synapses. Because μCaspian's run time depends on activity, we ran 1000 permutations of inputs selected uniformly at random on the μCaspian simulator and hardware and monitored the total number of spikes and the number of cycles used by the processor. μCaspian has a behaviourally accurate software simulator, and the hardware design can be emulated in Verilator or run on the FPGA. In this case, we used the UPdruino V3 as the FPGA board.

Over the 1000 runs, the simulator reported 73,159 total spikes for an average of 73 spikes per test case. Using Verilator, the 1000 test cases finished in $\sim$ 5,000,000 clock cycles, of which only $\sim$ 7000 cycles were used to load the virtual neuron network, and $\sim$ 5000 cycles were used per test case. Because the processor runs at 25 MHz, the total run time without the overheads from communication with the host computer is $\sim 0.21$ s for all test cases. When we ran the test using the UPduino FPGA, the total time was $\sim$ 400 s. One main culprit for this slowdown is the 3 MBaud UART connection between the host and the FPGA. While running on hardware, over $99.9\%$ of the execution time was spent in overhead and communication. This result highlights the great benefit of using the virtual neuron to perform addition on the SNN system instead of moving the data to a separate processor to perform the addition. The results from the hardware evaluation are tabulated in Table 7, and a summary of the μCaspian processor cycles from the experiment is in Table 8.

### mrDANNA power estimate

With neuromorphic application-specific integrated circuits, the power required for a particular network execution can be estimated based on the energy required for active and idle neurons and synapses for the duration of the execution. To estimate the power of the virtual neuron design, we used the same method and energy-per-spike values as reported by Chakma et al.^19^ for the mrDANNA mixed-signal, memristor-based neuromorphic processor. Using the same number of spikes, neurons, and synapses as reported in the μCaspian simulation, we estimate that a mrDANNA hardware implementation would use $\sim$ 23 nJ for the average test case run and around $\sim$ 23 mW for continuous operation.

**Table 7:** Summary of hardware evaluation.

Method	Execution time of processor	Wall time of evaluation	Power
μCaspian hardware	0.21 s	400 s	
μCaspian simulator	N/A	747 ms	
mrDANNA	1 μs @ 20 MHz	N/A	23.04 mW

**Table 8:** μCaspian cycles summary.

Entire test	
Clock cycles	5,000,000
Total time	0.21 s
Per test average	0.21 ms

Single test time	
Clock cycles	5000
Time	0.21 ms

Network load time	
Clock cycles	7000
Time	0.29 ms

## Applications

In this section, we look at five functions for which a virtual neuron is used: constant function, successor function, predecessor function, multiply by $-1$ function, and *N*-neuron addition.

### Constant function

**Figure 9:**
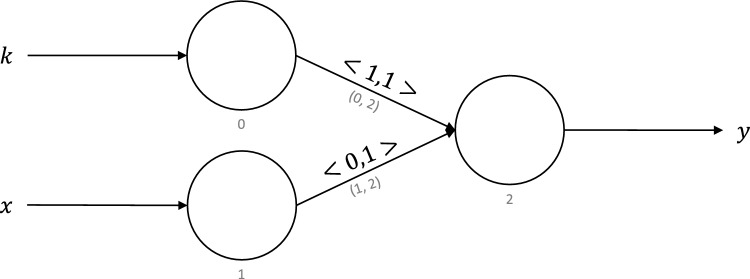
Neuromorphic circuit for constant function.

For a natural number, *x*, the constant function returns a constant natural number, *k*. It is defined as:

1 $$\begin{aligned} C_k(x) := k \end{aligned}$$

Figure 9 shows the neuromorphic circuit that computes the constant function. It has been adapted from previous work^13^ to work with the virtual neuron. Each neuron in this circuit is a virtual neuron. Note that virtual neurons 0 and 1 are used to convert the external input value with the encoding method used by the virtual neuron and to demonstrate that this behavior is possible in a larger network made from many virtual neurons. The same two-input virtual neuron definition can be used for these input neurons with the second input port disconnected.

Inputs *k* and *x* are fed to the input neurons 0 and 1, and the output is produced at neuron 2. Synapses going from neuron 0 to neuron 2 have weights of 1, whereas those going from neuron 1 to neuron 2 have weights of 0. The constant function is one of the $\mu$-recursive functions. The $\mu$-recursion is a model of computation that is equivalent to a Turing machine. To prove that a computing platform is Turing-complete, it must prove that it can execute all the $\mu$-recursive functions. With that in mind, being able to implement the constant function is a step toward empirically showing that neuromorphic computing is Turing-complete. We implemented the constant function circuit in NEST and tested it with 100,000 16-bit natural numbers generated uniformly at random. We were able to accurately execute the constant function using virtual neurons for all test cases. This run took 54,669 s for 100,000 cases in NEST, or roughly 0.5466 s per case.

### Successor function

**Figure 10:**
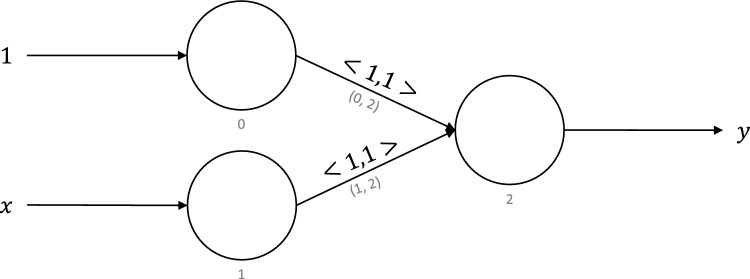
Neuromorphic circuit for successor function.

For a natural number, *x*, the successor function returns $x+1$. The successor of 0 is defined as 1. The successor function is defined as:

2 $$\begin{aligned} S(x) := x + 1 \end{aligned}$$

Figure 10 shows the neuromorphic circuit for the successor function. It too has been adapted from previous work^13^ and is another $\mu$-recursive function. It is similar to the constant function with a couple of differences. Neuron 0 is fed an input of 1, and synapse (1,2) has a weight of 1. We implemented the successor function using three virtual neurons in NEST and tested it on 100,000 16-bit numbers generated uniformly at random. Our implementation was able to execute the successor function successfully for all 100,000 test cases. This run took 54,657 s for 100,000 cases in NEST, or roughly 0.5466 s per case.

### Predecessor function

**Figure 11:**
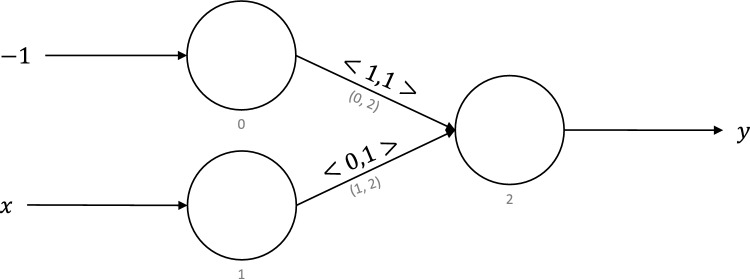
Neuromorphic circuit for predecessor function.

For a natural number, *x*, the predecessor function returns $x-1$. The predecessor function is defined as:

3 $$\begin{aligned} S(x) := x - 1 \end{aligned}$$

Figure 11 shows the neuromorphic circuit for the predecessor function. It is similar to the successor function with just one change. We feed an input of $-1$ to neuron 0 as opposed to 1. We implemented the predecessor function using three virtual neurons and tested it on 100,000 cases of 16-bit numbers generated uniformly at random. We were able to execute the predecessor function successfully using three virtual neurons in NEST for 100% of the test cases. This run took 54,243 s for 100,000 cases in NEST, or roughly 0.5424 s per case.

### Multiply by − 1

**Figure 12:**
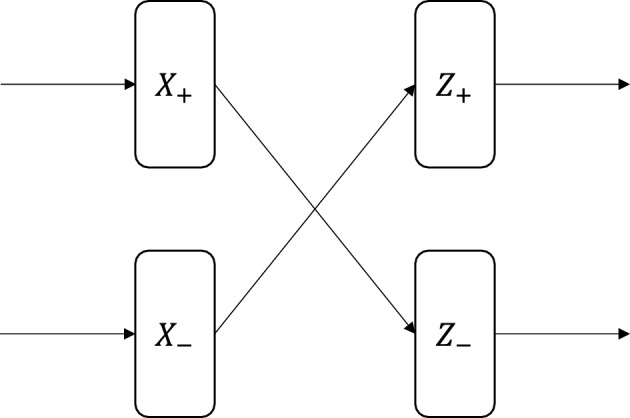
Neuromorphic circuit to multiply a number by $-1$.

For a rational number, *x*, this function returns $-x$. Figure 12 shows the neuromorphic implementation of the multiply by $-1$ function using the virtual neuron encoding to swap the interpretation and therefore the connection of the positive and negative bits of the number. It takes the rational number *X* encoded using the virtual neuron encoding method. In the figure, we use $X_+$ to denote the positive part of *X* and $X_-$ to denote negative parts of *X*. In this function, we assume that the number of positive and negative precision bits is equal. Under this assumption, we simply swap the positive and negative parts of *X* to return a number *Z* encoded using the virtual neuron method. Since $Z_+$ equals $X_-$, and $Z_-$ equals $X_+$, this function returns the negative of a number fed as the input. Note that this function affects the synaptic connections between virtual neurons. We implemented this function for 16-bit numbers in NEST and found that our virtual neuron-based implementation could execute the function successfully for all 100,000 test cases. This run took 39,361 s for 100,000 cases in NEST, or approximately 0.3936 s per case.

### N-neuron addition

**Figure 13:**
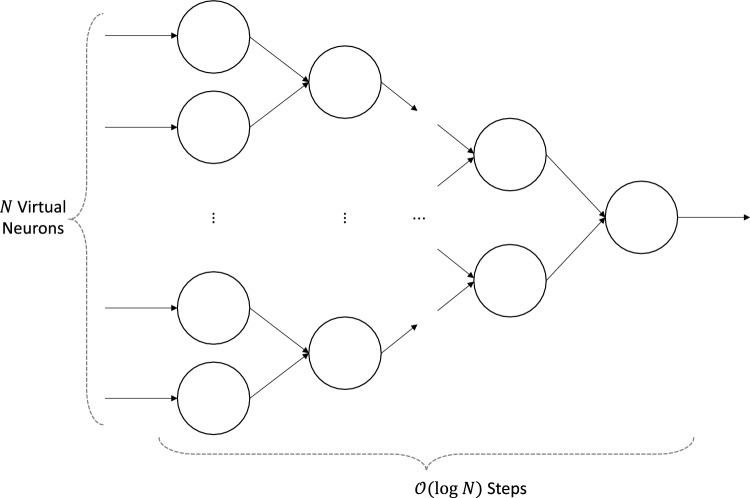
Neuromorphic circuit to add *N* virtual neurons.

The last application of the virtual neuron that we want to highlight is the *N*-neuron addition. The neuromorphic circuit for this application is shown in Fig. 13, where we would like to add *N* virtual neurons given as inputs. Here, the leftmost virtual neurons represent values using the virtual neuron encoding and could come from external input or from prior virtual neurons. The addition is performed by successfully connecting pairs of input virtual neurons to a layer of virtual neurons, which in turn serves as input to the next layer. This method uses $\mathscr {O}(N)$ virtual neurons and synapses and takes $\mathscr {O}(\log N)$ steps. Given that each addition operation in a virtual neuron takes $\mathscr {O}(N)$ time, the entire circuit in Fig. 13 takes $\mathscr {O}(N \log N)$ time. We implemented the *N*-neuron addition circuit in NEST and tested it on 100,000 cases of 16-bit numbers. This implementation successfully added *N* virtual neurons in $\mathscr {O}(N \log N)$ time in $100\%$ of the test cases. It took 114,295 s to run 100,000 cases in NEST, or approximately 1.14 s per case. This application shows that an arbitrary number of values could be added together by using a tree of virtual neurons.

## Discussion

In this paper, we proposed the virtual neuron as a mechanism for encoding as well as adding positive and negative rational numbers. Our work is a step toward a broader class of neuromorphic computing algorithms that would enable us to perform general-purpose computations on neuromorphic computers. In this paper, we also measured the time, space, and energy required for virtual neuron operations and showed that it takes around 23 mW of power for continuous operation. In addition to low operational power requirements, there are tremendous overhead savings when performing such operations within the spiking array without the need to spend time and energy sending the data to an external processor. Although it is beyond the scope of this paper, the virtual neuron is a vital component for composing subnetworks to scale-up neuromorphic algorithms, and it is also a vital component for network encoding and decoding capabilities. We would like to address these areas in future work.

The virtual neuron can be viewed as a higher-level abstraction that enables bit-level precision as well as variable precision on neuromorphic computers. We also expect the virtual neuron to be used in neuromorphic compilers that can compile high-level neuromorphic algorithms down to neurons and synapses and be deployed on neuromorphic hardware directly. Another potential use case of the virtual neuron is to encode and perform operations on extremely large numbers (containing thousands of bits), as is required in many cryptography applications. The virtual neuron would enable us to perform these large operations in an energy-efficient manner on neuromorphic computers.

In an edge computing scenario, current neuromorphic computers allow us to perform machine learning tasks by using SNNs in an energy-efficient manner. However, if an application requires general-purpose operations (e.g., for pre- or post-processing data using arithmetic, logical, and relational operations), then we resort to conventional computers (CPUs and GPUs), which incur significant communication cost. With approaches such as the virtual neuron, which serves as the backbone of general-purpose computing on neuromorphic computers, we could potentially perform all these operations on the neuromorphic computer itself without having to communicate to the CPU/GPU, thereby bypassing the need to transfer data back and forth from the CPU/GPU.

Notably, the ultimate goal of a neuromorphic computer may *not* be to perform these kinds of operations. However, many applications for which a neuromorphic system might be used (e.g., classification, anomaly detection, control) may require these types of calculations as a pre- or post-processing step for the neuromorphic system. For a continually operating neuromorphic system, for example in a control application, these sorts of calculations may be required *between* neuromorphic calculations. If these computations can take place *on* the neuromorphic computer, then it will alleviate communication costs and data movement to and from the neuromorphic system. As such, even if the computations described above are not as efficient as those on a traditional processor, it is likely that the data movement costs to and from the traditional processor will overwhelm the energy efficiency benefits gained from moving the computation back to a traditional processor.

Just like with IEEE standard data types, real numbers such as $\sqrt{2}, \pi$ and *e* cannot be directly encoded without infinite bits of precision. Therefore, the bits of precision used in the virtual neuron encoding can be chosen based on the accuracy of the approximation of the real number required. Lastly, the applications demonstrated in the “Applications” section might seem simple, but they are critical building blocks for any general-purpose computations that can be performed on a neuromorphic computer. Complex general-purpose compute tasks can be broken down into the simplest operations defined by these functions. We would like to reiterate that the goal of this paper was to present the idea of the virtual neuron and demonstrate its performance on physical and simulated neuromorphic hardware. The demonstrations described in “Applications” provide the reader with an idea of how the virtual neuron can be used.

## Conclusion

In this work, we presented the spike-based virtual neuron as a mechanism for encoding positive and negative integers and rational numbers. We implemented the virtual neuron in the NEST simulator and tested it on 8-, 16- and, 32-bit rational numbers. We compared the computational complexity of the virtual neuron to other neuromorphic encoding mechanisms. Next, we tested the virtual neuron on neuromorphic hardware and presented its time, space, and power metrics. Lastly, we demonstrated the usability of the virtual neuron by using it in five applications that would be crucial for general-purpose neuromorphic computing. We were able to show that the virtual neuron is an efficient mechanism for encoding rational numbers. Furthermore, we also showed that the virtual neuron can mimic the artificial neuron with an identity activation function.

Our work presents a key step in realizing the full potential of general-purpose neuromorphic computing by encoding numbers in a lossless manner beyond the currently supported Boolean or natural numbers. This would enable neuromorphic models to perform addition and multiplication in an exact manner and hence be used in regression, SVM, and graph algorithms. In our future work, we would like to explore general-purpose neuromorphic algorithms and applications using virtual neurons.

## Supplementary Information

Supplementary Information.

## Data Availability

The data that support the findings of this study are available from the corresponding author upon reasonable request.
