# Supplementary material for: Encoding integers and rationals on neuromorphic computers using virtual neuron
Source: Sci Rep. 2023 Jul 6;13:10975. doi: 10.1038/s41598-023-35005-x (PMC10326008; doi:10.1038/s41598-023-35005-x)
Supplement: Supplementary file 1 — Supplementary Information. [file 41598_2023_35005_MOESM1_ESM.pdf]

Virtual Neuron Implementation in Python using the NEST Simulator

```python
import nest

class VirtualNeuron:
  """ Virtual neuron is a neuromorphic primitive that mimicks the behavior of an artificial neuron using a collection of spiking neurons.
  """

  def __init__(self, precision=[4,4,4,4]):
    """ Initializes a virtual neuron object
    Params:
      precision: List of 4 ints denoting positive integer precision, positive fractional precision, negative integer precision and negative fractional precision
    """

    self.precision = precision
    self.positive_precision = precision[0] + precision[1]
    self.negative_precision = precision[2] + precision[3]
    self.total_precision = sum(precision)
    self.higher_precision = max(self.positive_precision, self.negative_precision)


    # Setup incoming neurons
    self.x_positive = {}
    self.x_negative = {}
    self.y_positive = {}
    self.y_negative = {}

    for i in range(self.positive_precision):
      self.x_positive[i] = self.create_neuron(threshold=0)
      self.y_positive[i] = self.create_neuron(threshold=0)

    for i in range(self.negative_precision):
      self.x_negative[i] = self.create_neuron(threshold=0)
      self.y_negative[i] = self.create_neuron(threshold=0)


    # Setup positive bit neurons
    self.bits_positive = {}
    if self.positive_precision > 0:
      for i in range(self.positive_precision + 1):
        self.bits_positive[i] = {}
        self.bits_positive[i][0] = self.create_neuron(threshold=0)
        self.bits_positive[i][1] = self.create_neuron(threshold=1)

        if i > 0:
          self.bits_positive[i][2] = self.create_neuron(threshold=2)


    # Setup negative bit neurons
    self.bits_negative = {}
    if self.negative_precision > 0:
      for i in range(self.negative_precision + 1):
        self.bits_negative[i] = {}
        self.bits_negative[i][0] = self.create_neuron(threshold=0)
        self.bits_negative[i][1] = self.create_neuron(threshold=1)

        if i > 0:
          self.bits_negative[i][2] = self.create_neuron(threshold=2)


    # Setup outgoing neurons
    self.z_positive = {}
    self.z_negative = {}

    if self.positive_precision > 0:
      for i in range(self.positive_precision + 1):
        self.z_positive[i] = self.create_neuron(threshold=0)
```

```
    if self.negative_precision > 0:
      for i in range(self.negative_precision + 1):
        self.z_negative[i] = self.create_neuron(threshold=0)


    # Neurons created
    print("Neurons created...")


    # Setup synapses between positive incoming neurons and positive bit neurons
    for i in range(self.positive_precision):
      nest.Connect(self.x_positive[i], self.bits_positive[i][0], syn_spec={"weight": 1.0, "delay": float
    (i+1)})
      nest.Connect(self.x_positive[i], self.bits_positive[i][1], syn_spec={"weight": 1.0, "delay": float
    (i+1)})
      nest.Connect(self.y_positive[i], self.bits_positive[i][0], syn_spec={"weight": 1.0, "delay": float
    (i+1)})
      nest.Connect(self.y_positive[i], self.bits_positive[i][1], syn_spec={"weight": 1.0, "delay": float
    (i+1)})

      if i > 0:
        nest.Connect(self.x_positive[i], self.bits_positive[i][2], syn_spec={"weight": 1.0, "delay":
    float(i+1)})
        nest.Connect(self.y_positive[i], self.bits_positive[i][2], syn_spec={"weight": 1.0, "delay":
    float(i+1)})


    # Setup synapses between negative incoming neurons and negative bit neurons
    for i in range(self.negative_precision):
      nest.Connect(self.x_negative[i], self.bits_negative[i][0], syn_spec={"weight": 1.0, "delay": float
    (i+1)})
      nest.Connect(self.x_negative[i], self.bits_negative[i][1], syn_spec={"weight": 1.0, "delay": float
    (i+1)})
      nest.Connect(self.y_negative[i], self.bits_negative[i][0], syn_spec={"weight": 1.0, "delay": float
    (i+1)})
      nest.Connect(self.y_negative[i], self.bits_negative[i][1], syn_spec={"weight": 1.0, "delay": float
    (i+1)})

      if i > 0:
        nest.Connect(self.x_negative[i], self.bits_negative[i][2], syn_spec={"weight": 1.0, "delay":
    float(i+1)})
        nest.Connect(self.y_negative[i], self.bits_negative[i][2], syn_spec={"weight": 1.0, "delay":
    float(i+1)})


    # Setup carry synapses in positive bits
    for i in range(self.positive_precision):
      nest.Connect(self.bits_positive[i][1], self.bits_positive[i+1][0], syn_spec={"weight": 1.0, "delay
    ": float(1.0)})
      nest.Connect(self.bits_positive[i][1], self.bits_positive[i+1][1], syn_spec={"weight": 1.0, "delay
    ": float(1.0)})
      nest.Connect(self.bits_positive[i][1], self.bits_positive[i+1][2], syn_spec={"weight": 1.0, "delay
    ": float(1.0)})


    # Setup carry synapses in negative bits
    for i in range(self.negative_precision):
      nest.Connect(self.bits_negative[i][1], self.bits_negative[i+1][0], syn_spec={"weight": 1.0, "delay
    ": float(1.0)})
      nest.Connect(self.bits_negative[i][1], self.bits_negative[i+1][1], syn_spec={"weight": 1.0, "delay
    ": float(1.0)})
      nest.Connect(self.bits_negative[i][1], self.bits_negative[i+1][2], syn_spec={"weight": 1.0, "delay
    ": float(1.0)})


    # Setup synapses between positive bit neurons and positive outgoing neurons
    if self.positive_precision > 0:
      for i in range(self.positive_precision + 1):
        nest.Connect(self.bits_positive[i][0], self.z_positive[i], syn_spec={"weight": 1.0, "delay":
```

```
    float(self.higher_precision - i + 1)})
118         nest.Connect(self.bits_positive[i][1], self.z_positive[i], syn_spec={"weight": -1.0, "delay":
    float(self.higher_precision - i + 1)})
119
120         if i > 0:
121           nest.Connect(self.bits_positive[i][2], self.z_positive[i], syn_spec={"weight": 1.0, "delay":
    float(self.higher_precision - i + 1)})
122
123
124     # Setup synapses between negative bit neurons and negative outgoing neurons
125     if self.negative_precision > 0:
126       for i in range(self.negative_precision + 1):
127         nest.Connect(self.bits_negative[i][0], self.z_negative[i], syn_spec={"weight": 1.0, "delay":
    float(self.higher_precision - i + 1)})
128         nest.Connect(self.bits_negative[i][1], self.z_negative[i], syn_spec={"weight": -1.0, "delay":
    float(self.higher_precision - i + 1)})
129
130         if i > 0:
131           nest.Connect(self.bits_negative[i][2], self.z_negative[i], syn_spec={"weight": 1.0, "delay":
    float(self.higher_precision - i + 1)})
132
133
134     # Synapses created
135     print("Synapses created...")
136     print("Virtual neuron created...")
137
138
139   def create_neuron(self, V_th=0, internal_state=-1.0):
140     """ Creates an "iaf_psc_delta" neuron in NEST
141     Params:
142       V_th: Threshold voltage
143       internal_state: Default internal state of the neuron
144
145     Returns:
146       neuron: A NEST neuron of type iaf_psc_delta
147     """
148
149     neuron = nest.Create("iaf_psc_delta")
150     neuron.V_th = V_th
151     neuron.V_m = internal_state # Membrane potential
152     neuron.V_reset = -1e-6
153     neuron.tau_m = 1e-6   # Leak
154     neuron.t_ref = 0.0
155     neuron.E_L = neuron.V_m # Resting membrane potential
156     refractory_input = False
157     return neuron
```
